# Proteomics, lipidomics, metabolomics: a mass spectrometry tutorial from a computer scientist's point of view

**DOI:** 10.1186/1471-2105-15-S7-S9

**Published:** 2014-05-28

**Authors:** Rob Smith, Andrew D Mathis, Dan Ventura, John T Prince

**Affiliations:** 1 Department of Computer Science, Brigham Young University, 84606 Provo, USA; 2 Department of Chemistry, Brigham Young University, 84606 Provo, USA

## Abstract

**Background:**

For decades, mass spectrometry data has been analyzed to investigate a wide array of research interests, including disease diagnostics, biological and chemical theory, genomics, and drug development. Progress towards solving any of these disparate problems depends upon overcoming the common challenge of interpreting the large data sets generated. Despite interim successes, many data interpretation problems in mass spectrometry are still challenging. Further, though these challenges are inherently interdisciplinary in nature, the significant domain-specific knowledge gap between disciplines makes interdisciplinary contributions difficult.

**Results:**

This paper provides an introduction to the burgeoning field of computational mass spectrometry. We illustrate key concepts, vocabulary, and open problems in MS-omics, as well as provide invaluable resources such as open data sets and key search terms and references.

**Conclusions:**

This paper will facilitate contributions from mathematicians, computer scientists, and statisticians to MS-omics that will fundamentally improve results over existing approaches and inform novel algorithmic solutions to open problems.

## Background

Robust data processing tools for MS data are lagging behind the substantial advances occurring in instrumentation and protocol [1]. One reason for this is that few outside experts--mathematicians, computer scientists, and statisticians--have climbed the learning curve (usually requiring several years of dedicated study) to understand the terminology, chemical theory, workflows, and challenges of MS-omics (proteomics, lipidomics, and metabolomics). This sort of interdisciplinary learning curve is not unusual in bioinformatics; however, the influx of external experts to genomics has not been seen to date in MS-omics. One reason for this is the lack of a succinct and cogent introductory resource that can bring outside experts to a basic but functional level of MS-omics familiarity.

In this primer, we will elucidate the mechanisms of MS-omics, the problems it is used to solve, key concepts and terms found in the literature, and open problems and their salient literature. The purpose of this tutorial is to expedite the new researcher's acquisition of a functional knowledge of MS-omics sufficient for contribution to the field.

## Results and discussion

### Relationship of genomics, proteomics, lipidomics, and metabolomics

The exponential growth of genomics studies during the last ten years has not been matched by corresponding research in MS-omics [2]. Genomics researchers have several peer-reviewed conferences in which to publish their results. To the best of our knowledge, there has not been a single peer-reviewed conference to date on lipidomics or metabolomics, let alone any specifically addressing algorithmic approaches to problems specific to either area, although there are periodic special genomics conferences dedicated to proteomics. Several existing venues labeled as bioinformatics will not accept papers on MS-omics, as their stated area of interest is limited to a distinct subfield of bioinformatics such as genomics. This phenomenon of focus on genomics is also reflected in institutional research programs. In a recent review of 78 post-secondary degree-granting bioinformatics programs, 22 programs noted a research emphasis in genomics, while 18 noted a research emphasis in proteomics. Not a single institution listed a research program in lipidomics or metabolomics [3].

The biological reach and impact of research in MS-omics is so extensive that it can be argued that MS-omics should now be the highest priority of systems biology [4]. From a pragmatic perspective, the large set of fresh problems and substantial potential for impact in MS-omics ought to be very attractive to those in more crowded disciplines.

#### Proteomics

Proteomics is the study of biological processes via the analysis of protein expression or state in cells or tissue. Proteins are ubiquitous building blocks of life, and they are composed of peptides, which are chains of amino acids built by translating mRNA. There are 20 amino acids, uniquely abbreviated with a single letter. Peptides thus can be described as a string of the letters corresponding to the amino acids. Though protein sequences are determined by DNA sequences, post translational protein modifications (such as acetates, phosphates, lipids etc.) are not as easily predicted. These modifications quickly diversify and regulate/complicate protein function and cellular protein composition and are characteristic in most cellular processes and diseases. Therefore, the aim of MS-proteomics is to provide data that DNA sequences cannot--namely, individual protein concentrations and identification of post-translational modifications.

#### Lipidomics

Lipidomics is the systems-level analysis of lipids (fat molecules) and their interactions [5]. It is a science still in its infancy but one that promises to revolutionize biochemistry [4]. Lipids are grouped into eight categories that share common physical and chemical properties [4,6], and there are currently some 38,000 documented lipids.

Lipids that occur rarely or in small quantities are often the most effectual lipids in biological processes, meaning they are particularly important in disease diagnostics and in understanding pathology [5]. Lipidomics can elucidate the pathology and treatment of many diseases such as cancer, diabetes, obesity, cardiovascular disease, arthritis, asthma, inflammatory bowel disease, Alzheimer's and others due to the associated disruption of lipid metabolic enzymes and pathways [7,2,8,5]. A better understanding of lipidomics could significantly advance diagnostic medicine as well as provide novel treatment options.

#### Metabolomics

Metabolomics is the study of metabolomes--small molecular end products of cellular regulatory pathways [9] that can provide a snapshot of cell physiology. Metabolites are much smaller than proteins and smaller than most lipids. Their small size precludes the direct overlap of some techniques used in proteomics or lipidomics, but they may be generally analyzed in similar ways. Lipids may be classified as a subset of metabolites; however, mass spectrometrists typically consider lipids distinct from metabolites because analytically they must be treated separately (i.e., require different solvents).

### MS-omics pipeline

**Figure 1:**
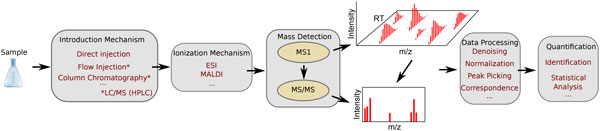
**The MS-omics pipeline**. A sample is introduced to an ionization mechanism with or without a preliminary separation technique, where particles receive a charge enabling the detector to estimate the mass-to-charge ratio (m/z) and intensity of each analyte. If the system has tandem mass spec capabilities, some precursor ions (MS1) are selected for fragmentation (MS/MS). Data processing techniques prepare the data to be quantified via statistical methods and identified via matches to theoretical databases.

The workflow from sample preparation to result quantification, can be split into two consecutive pipelines: the wet-lab pipeline and the data processing pipeline. The data processing pipeline consists of many possible processing steps that take the data resulting from the wet-lab pipeline (the mass spectrometer output) to the end result: identification and quantification (see Figure 1). The quality of each step in the pipeline affects the sensitivity and reliability of the outcome [10]. There are many optional steps, some of them very popular. We will describe the essential and some optional steps.

**Figure 2:**
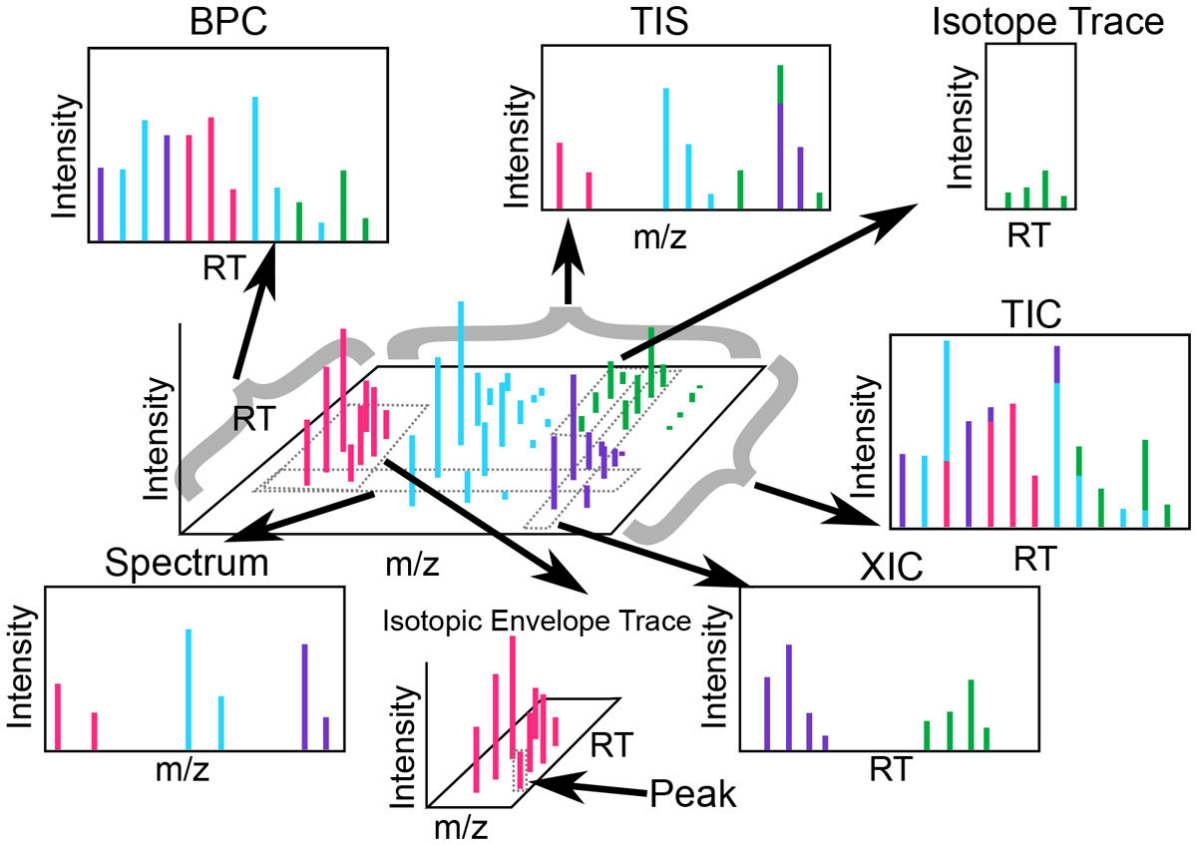
**Common nomenclature**. Each portion or summary of an MS run is referred to by a different name. A *spectrum *contains all points with a single RT value. The sum of signals across all spectra is called the *total ion spectrum (TIS)*. A slice of data containing a contiguous m/z range extending across all RT is called an *extracted ion chromatogram (XIC)*. While the *total ion chromatogram (TIC) *is the sum of all signals across all m/z, the *base peak chromatogram (BPC) *is the set containing the most intense signal for each RT across all m/z. An *isotope trace *is the signal produced by a single ion of a single analyte (i.e., a peptide or a lipid) at a particular charge state. An *isotopic envelope trace *is the group of isotopic traces produced by a single analyte at a particular charge state. Note that certain terms like peak, feature, and chromatogram, are overloaded in the literature and as such are exceedingly unclear.

All MS experimental data share a set of descriptive keywords that are essential for referencing components of the output map (see Figure 2). A comprehensive reference of key MS terms is provided in [11].

#### Sample preparation

The details of sample preparation are beyond the scope of this paper. However, at a general level, sample preparation strategies prior to mass spectral analysis are based on isolating analytes of interest and removing all other contaminating molecules. For instance, filters can be used to separate high molecular weight proteins from low molecular weight lipids and metabolites, or contaminates. Other sample preparation techniques exploit analyte hydrophobicity, charge, and analyte-specific affinity. The degree of specificity in sample preparation is determined by the end goal of the experiment [12]. For example, if an experiment requires the analysis of only phosphorylated proteins, the sample preparation should isolate only phosphorylated proteins. Of course, this is very challenging but using an appropriate sample preparation strategy specific to an experimental need significantly simplifies mass detection and data analysis and in some cases is required to identify analytes of interest. Proteomics, lipidomics, and metabolomics each have unique considerations in sample preparation.

#### Introduction methods

Direct injection refers to infusing the sample directly into the mass detector. This is usually done with some sort of machine to make the flow constant.

While it is sometimes advantageous to allow all analytes to flow through detection at once, most MS experiments of complex samples will use chromatography due to its ability to spread out analytes over time, making it less likely that the ionization capacity will be overcome by large quantities of analyte or background ions, a phenomenon called ion suppression.

Chromatography disperses the introduction of analytes into the mass detector through time based on some chemico-physico property (hydrophobicity, for instance). All chromatography systems have two phases: the stationary phase and the mobile phase. The stationary phase causes analyte separation and the mobile phase carries the analytes through the chromatographic column to the mass spectrometer. Methods include:

• LC-MS - mass spectrometry coupled to liquid chromatography. Liquid chromatography uses a liquid mobile phase and a column packed with chemically derivated beads as a stationary phase. The mobile phase is composed of a two-liquid gradient. Changes in the gradient (the percent composition of each liquid) cause analytes to be slowly released from the column and enter the mass spectrometer. Different stationary phases can separate analytes based on hydrophobicity, charge, size, or affinity. However, the most common stationary phases for LC-MS on biomolecules are reversed phase (hydrophobic) and strong cation (charge) [13].

• GC-MS - mass spectrometry coupled to gas chromatography. In gas chromatography systems the mobile phase is an inert gas (such as helium) and the stationary phase is a column designed to separate molecules based on polarity. The gradient is temperature increase; molecules with a high affinity for the column elute at higher temperatures.

• CE-MS - mass spectrometry coupled to capillary electrophoresis. Electrophoresis differs from chromatography, relying on electric fields, rather than mobile and stationary phases, to separate molecules [14]. Capillary electrophoresis uses an electric field applied to long narrow capillaries to separate molecules based on size, charge, and flow resistance through the capillary.

Multidimensional chromatography (sometimes referred to as tandem chromatography) refers to two chromatographic systems applied to the same system. In the case of LC-GC-MS, for example, analytes are introduced into the gas chromatography system as they elute from the LC system, with each system causing analytes with specific properties to elute with precedence. A more common multidimensional system in MS-omics is MUDPIT. MUDPIT uses two orthogonal separation strategies like strong cation ion exchange (charge based) and reversed phase (hydrophobicity based) chromatography to achieve greater resolution.

#### Ionization methods

Analytes must be ionized (i.e., in a charged state) in order to be detected by the mass spectrometer. Electrospray ionization (ESI) was developed in 1994 and is the most popular in MS-omics due largely to its ability to ionize unstable molecules without breaking chemical bonds and to the diverse range of analytes that can be ionized by the method [15,16]. Other methods include atmospheric pressure chemical ionization (APCI) [17], matrix-assisted laser/desorption ionization (MALDI) [17], and electron-ionization (EI) [17]. Ionization methods for ms-omics are generally referred to as soft ionization methods and include ESI and MALDI. EI is a harsh ionization method and will destroy most biomolecules except for very stable lipids and metabolites.

#### Mass detection

As charged particles are passed through the mass spectrometer, the mass-to-charge ratio (m/z) of detected particles is registered. A single scan on the resulting output represents a snapshot of the precursor ions passing through the mass spectrometer at that particular retention time (RT). The ions in this stage are called precursor ions because in tandem mass spectrometry (MS/MS), ions in small m/z windows are captured for fragmentation and MS detection a second time, yielding a second set of ions called product ions that can be used to identify precursor ions by matching their MS/MS patterns to a database of possibilities. It is important to understand that the ratio of solution selected for MS/MS fragmentation is low, normally capturing only 10-20% of the precursor (MS1) data. Because most MS/MS systems autoselect what segments to capture based on intensity, much of that portion overlaps between replicates. Of that 10-20%, less than 60% are identified via database lookup, and even that is subject to false positive identifications [18].

An analyte can contain certain naturally occurring rare isotopes, such as carbon-13. These isotopes tend to occur in individual analytes in known quantities, causing a characteristic pattern called an isotopic envelope (see Figure 2). The envelope is characterized by the number of and relative intensity between its isotopes. The monoisotopic peak, or peak that appears at the theoretical mass discounting any attached heavy isotopes, usually appears alongside the slightly heavier masses of any portion of the peptide or lipid in the sample that contains heavy isotopes.

**Figure 3:**
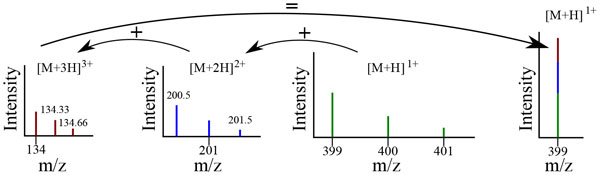
**Deisotoping**. A contrived example of deistoping. The same molecule is displayed here in three reduced isotopic envelopes (denoted by color) created from single- ([*M *+ *H*]^+1^), double- ([*M *+ *H*]^+2^) and triple-charged ([*M *+ *H*]^+3^) instances of the molecule. The monoisotope (the lowest m/z ion) from each isotopic envelope is combined to form the deistoped monoisotopic peak.

When an analyte exists in a run in more than one charge state (a very common occurrence due to variability in ionization), its isotopic envelope will reappear in a compressed and shifted form due to increased charge, as illustrated in Figure 3. The equation for the shift is specific to the source of the charge. For instance, a charge can be induced by the addition of a proton, in which case the shift is defined by (*µ *+ *k*)/charge m/z with a gap between ions in the isotopic envelope of 1/*k*, where *k *is the charge of the analyte (3+, 2+, 1+, and 1+, respectively in Figure 3) and *µ *is the m/z of the single-charged analyte (this is the analyte with only a +1 charge--399 in Figure 3).

**Figure 4:**
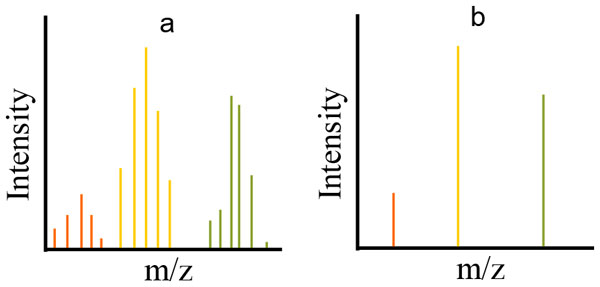
**A profile (a) and centroid (b) version of the same spectrum**. The profile raw data detected by a mass spectrometer consists of distributed signal across m/z values at each point where an ion is detected. Centroid data is raw data that has been processed by an algorithm to retain only the local maximum in each range in which an ion is detected. Because each ion detected creates an m/z distribution of signal, the distribution itself (in profile mode) or the maximum to which it is reduced (its centroid) is sometimes called a peak. This ion intensity distribution along m/z is not to be confused with the distribution of ion intensity along time in chromatographic studies (see Figure 2).

Mass spectrometers output raw data--a large collection of data points each consisting of a tuple of m/z, intensity, and time (RT) either in profile or centroid form. Profile data contains all data points registered by the mass spectrometer (see Figure 4a), while centroid data has been reduced to data points that represent the local maxima in a single spectrum, a distribution of data over an m/z range for a given RT (see Figure 4b). Centroid data is much more concise than profile data, but the reduction incurs information loss.

Experiments can run in full scan mode--where the full range of m/z values is read--or the mass spectrometer can scan only certain m/z values (called single reaction monitoring in the case of one m/z value or multiple reaction mode in the case of several) [17].

Mass spectrometers have varying characteristics depending on the mechanisms used for mass detection, each with a different resolution. Resolution at a certain m/z is given by the ratio of that m/z to the smallest m/z gap between two distinguishable ions. Higher resolution instruments yield narrower profile peaks (see Figure 4a), allowing the signals from two distinct ions to be distinguished despite their similarity in m/z.

#### Data processing

Data processing consists of each of the possible steps in the MS-omics pipeline (Figure 1) involving digital manipulation of the mass spectrometer data or products from that data. These methods are constantly being improved upon and are discussed in detail in Section. Here, we provide a high-level overview of the role of data processing in the MS-omics pipeline.

The first step in data processing is handling the raw data produced by the mass spectrometer. Algorithms for noise reduction, feature detection, and correspondence exist that operate on the raw data. However, many require preliminary conversion out of the proprietary data format of the instrument and into an open data type (see below for a discussion of existing data types). It is important to note that, due to the size of the data sets, random access data processing--where only a portion of the data file is loaded into memory at a time--is a must, although some current tools load the full file and are therefore prone to crashing and subject to file size limits as memory is exhausted.

Prior to analyte identification, the data must be denoised, peak-picked, featuredetected, deisotoped, and deconvoluted. These are significant and open problems and are discussed in more detail below.

Analyte identification follows data processing. Here, one of several available databases are used to compare the experimental feature observations (i.e. isotopic envelopes, isotopic traces, etc.) to theoretical patterns. These include Sequest [19] for proteins, LIPIDMAPS [20] for lipids, and METLIN [21] for metabolites. Due to incomplete/growing databases and noisy data, closest-match assignment is prone to false positives and mismatches. Statistical analysis is almost always incorporated in this or prior steps in order to ascertain the significance of the identification.

The ultimate goal of data processing is to yield the quantity of each analyte. The identification and quantity of analytes, as well as the underlying raw data, must be stored in data structures that allow for efficient access and manipulation of the data.

### Data types

Raw data is a general label that actually describes a set of data formats specific to the vendor of the instrument. Many data converters from raw to open data formats exist. One popular converter is pwiz (http://proteowizard.sourceforge.net/). The Network Common Data Form (NetCDF), a generic open science data format, is an early data format that is still in use in some applications. mzXML is an open XML based data format with wide support. mzML was developed to replace mzXML and has more information from the raw data encoded and uses extensible ontologies to encode meta-data. mzQuantML is an open data format specifically intended for the storage of quantities associated with identified feature data. mzIdentML and pepXML are standards designed to facilitate database identity searches. Annotated Putative Peptide Markup Language (APML) is an XML standard designed to provide a single data file encoding of the original data set and its modifications via data processing tools [22].

### Data sets

#### Lack of labeled data

The prevailing problem in developing and evaluating computational approaches to MS-omics problems is the lack of labeled data [23]. Labeled data is difficult to obtain both because of the size of data sets--which can easily consist of millions of data points per file and hundreds of GBs of files for a replicate experiment series--and the undependability of hand-labeling--which is both time consuming and subjective. Several approaches for mitigating this problem exist: qualitative metrics, spiked mixtures, and *in silico *simulated data.

*Qualitative metrics *Evaluation metrics that do not use ground truth avoid the need for labeled data. For example, replicate alignment quality can be assessed via the Pearson correlation coefficient, feature overlap rate, or coefficient of variation. This approach is sub-optimal, as a good score on a qualitative metric does not necessarily translate into a good quantitative score using labeled data, but it is easy to compute and is comparable across problem instances.

*Spiked mixtures *Commercially available purified and quantified measures of a specific analyte are combined to produce a data set with known composition and quantity. These samples are not exactly ground truth, however. Due to ionization inefficiencies, environmental contaminants, and the variability of mass spectrometry, no instrument will report the same quantity and composition predicted by a spiked mixture. What's more, a mixture of a few analytes, which often do not co-occur in nature, is hardly representative of real-world scenarios, in which complex samples can easily contain hundreds of thousands of distinct analytes. To create more realistic conditions, spiked mixtures can be added to samples where the spiked an-alytes are not expected to occur. However, a method's accuracy on a few analytes is not necessarily indicative of performance across all analytes, particularly given the variability and limitations of MS/MS, which is commonly used to single out the m/z of the expected analytes but cannot be expected to capture the gross majority (≈ 80 − 90%) of the remaining sample.

In silico *simulated data *Simulated data is used in the field to refer to real-world data sets that have been purtubed with m/z shifts or intensity value modifications in order to create psuedo-new data without having to rerun costly experiments. True simulated data, called *in silico *to identify that it as purely sourced from simulation algorithms on a computer, is a relatively new advent in MS-omics. Creating realistic *in silico *data requires the analysis of many ground truth datasets, which creates a chicken and egg problem, as the difficulty of obtaining ground truth datasets is the very reason an *in silico *simulator would be beneficial.

#### Sources of open data

To facilitate strictly algorithmic advances in MS-omics, to avoid the need for a costly wet lab for creating mass spectrometry data, and to aid in evaluative comparisons against existing methods, more and more practitioners are making their data freely available online. Although any serious foray into MS-omics should certainly include a collaborator with mass spectrometry assets and formal training, we present a list of some of these open data sets in order to aid those who are interested in investigating MS-omics for the first time as well as more seasoned investigators who would simply like to make a case for the generality of their methods.

Lange *et al*. have provided two proteomic and two metabolomic data sets [24] which they have used to assess the quality of several alignment algorithms at http://msbi.ipb-halle.de/msbi/caap. The data is already segmented into reduced isotopic envelopes (isotopic envelopes whose isotopic traces are integrated into a single point).

Listgarten *et al*. provide centroided replicate data with spiked-in peptides [25]. There are two data sets: a set of 11 replicate LC-MS runs from ruptured *E. Coli *cells and a set of 14 LC-MS runs of human serum samples.

Jeffries provides a data set consisting of raw replicates of SELDI data [26] at http://data.ninds.nih.gov/Jeffries/alignment/index.html.

The SuperHirn data set [27] can be found at http://proteomics.ethz.ch/muellelu/web/Latin_Square_Data.php. It consists of 18 LC-MS runs from tryptic digests of 6 nonhuman proteins spiked with different concentrations into a complex human peptide sample and includes the raw as well as processed data. The data was obtained on an FT-LTQ.

### Problems of interest

Among the data processing portion of the MS-omics pipeline, some problems are widely studied, and some are emerging. All provide future research potential.

#### In silico *simulation*

The lack of ground truth data for evaluation of data processing algorithms precludes effective validation and comparison. *In silico *data simulation is a relatively new approach to providing on demand ground truth simulated data. By modeling a list of analytes and a description of experimental conditions, simulators can provide estimates of mass spectrometer output combined with labels of the analytes and quantities used *in silico *to generate the data (see [28-31]).

#### Correcting mass shift

Analyte detection on the m/z axis in mass spectrometers is subject to two types of error: systematic mass error--a functional deviation from true mass--and random mass error [32]. Typically, systematic mass error is mitigated by routine machine recalibration--a process wherein analytes of known mass are processed in the mass spectrometer to create a model that is used to interpolate m/z shift for any given m/z value. However, the efficacy of this calibration reduces over time as the mass constantly continues to shift. Additionally, some machines benefit from an injection of spiked standards during a normal experiment for internal calibration, which helps overcome the temporal effects of space charge effects, electric fields, peak intensity, and temperature [32]. Internal standards are undesirable due to the additional cost of standards and the suppression implications of spiked standards. Computational mass calibration techniques have been proposed in order to provide the mass accuracy of internal calibration but with better consistency and lower cost [32]. This is an active but not crowded area of research with practical implications.

#### Correspondence

**Figure 5:**
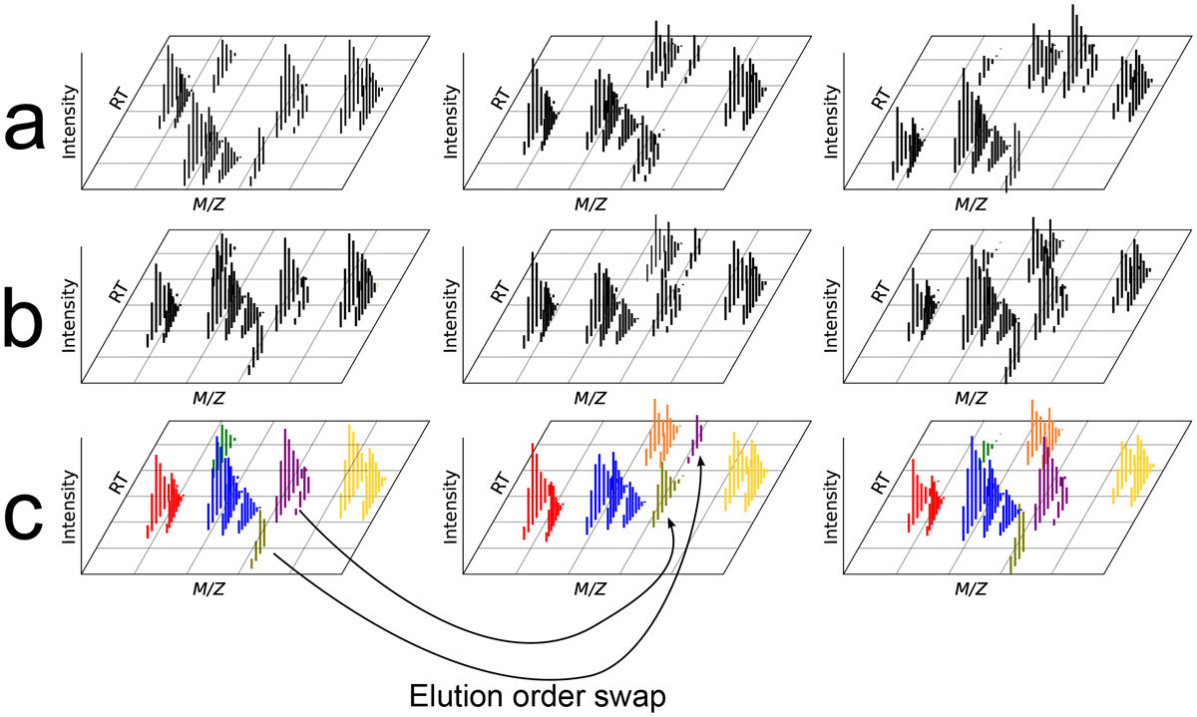
**MS correspondence**. Correspondence is the problem of registering features across multiple samples (matches across the samples are depicted in the same color). Most times this process is facilitated by aligning the retention time (RT) of features across multiple samples (top to bottom row). Note that features are almost never present across all samples and can display significant RT variability and (to a lesser degree) m/z variability.

Correspondence, the registration of recurring signals from the same analyte over replicate samples, is a crucial problem in any of the many MS experiments where multiple runs of similar samples are compared to each other (see Figure 5). For a comprehensive review of current algorithms, see [33]. Persisting problems are an abundance of user parameters, models that do not include known behavior, prohibitively long runtimes, and a lack of performance comparison between methods [34].

#### Denoising

**Figure 6:**
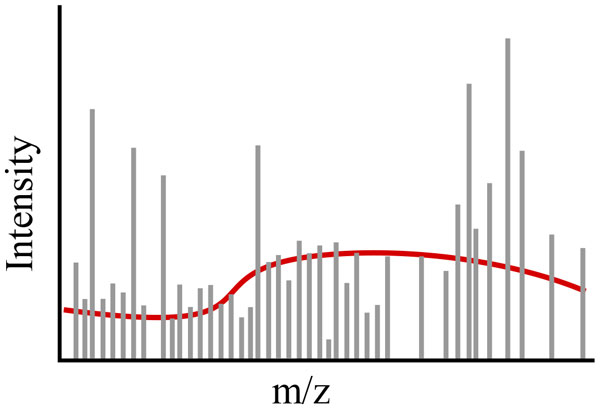
**Baseline subtraction**. Baseline subtraction is the functional estimation and removal of background noise.

MS-omics produces inherently noisy data. Noise can consist of spurious data points or distortion of a data point's true value in retention time, m/z, or intensity. Denoising as used in MS-omics refers to the removal of spurious data points. Baseline subtraction is a common method in which signals with intensity lower than an adaptive threshold are considered to be noise and removed (see Figure 6). This is an active area of research, though most experiments in the literature have not made an explicit and dedicated study of different techniques, instead describing the denoising method applied as a data processing step in a larger experiment.

#### Feature detection

**Figure 7:**
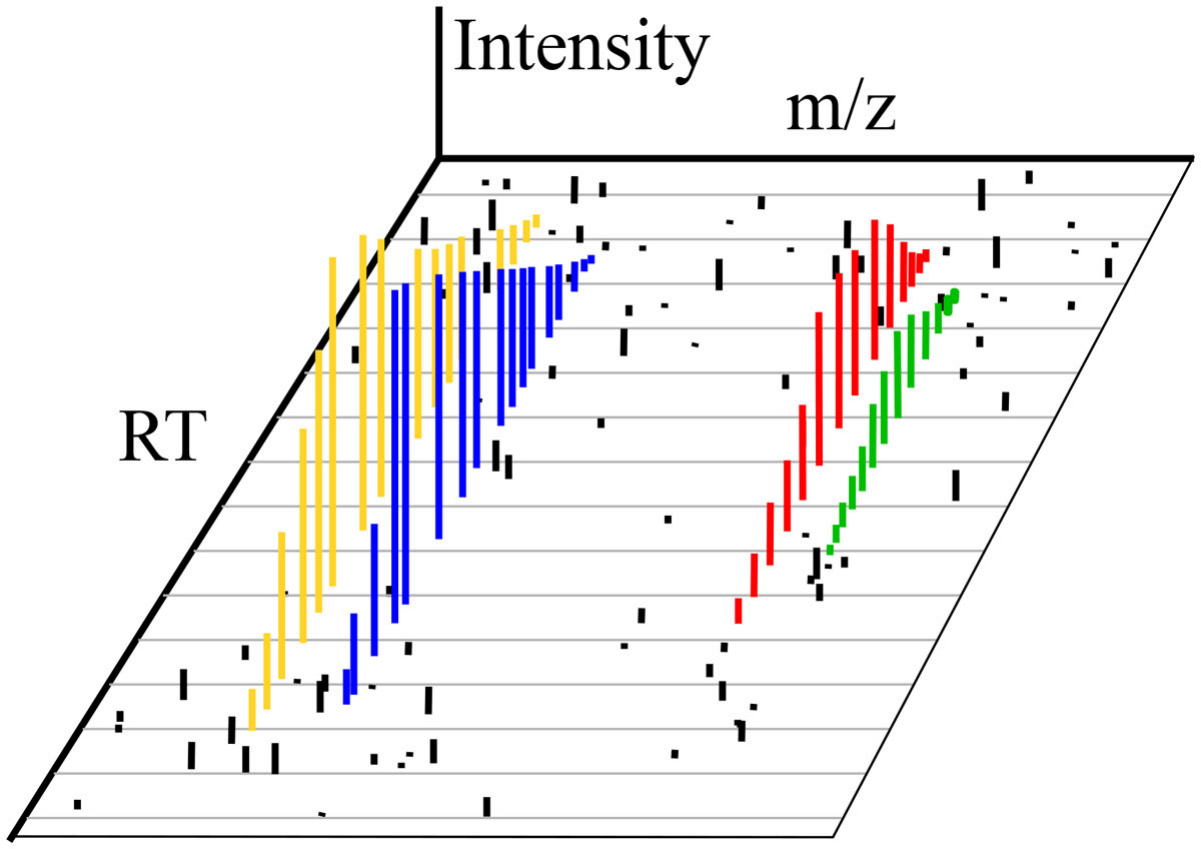
**Feature detection**. Feature detection consists of labeling data points which pertain to individual features (indicated by color here) while excluding noise points (in black).

The most important step of an MS-omics workflow is undoubtedly feature detection [1], a general term that can apply to the extraction of various signal elements from MS data. In chromatographic data, feature detection can refer to either extracting isotopic envelopes or isotopic traces from an MS sample output (see Figure 7). Many methods exist for isotope trace extraction, among them a promising new algorithm that performs well on existing evaluations [35]. Sometimes this process is called peak picking or peak detection, but those terms should be avoided since they are also used to refer to the conversion from profile data to centroid data. In direct injection data, feature detection is sometimes referred to as peak summarization, since each spectra (being an approximation of the latent content of the non-chromatographically separated sample) must be combined into a TIS through mitigating the variance inherent in m/z across spectra (see [36]).

#### Data structures

As described earlier, many data types exist for MS-omics data. New data formats continue to be proposed to meet unforeseen needs.

A recent prevailing expansion point has been the need to store the results of data processing tools in addition to the original data. Truly modular pipelines require data structures that contain all necessary data to be used by any tool in the pipeline, meaning previous modifications are annotated in addition to retention of the original data. APML is one attempted solution to this problem, but, so far, the community has not embraced it, as it appears that there are only two extant algorithms which use it [22].

There is still a need for compact, random access, and information rich data structures and access for MS data [37]. What's more, some proprietary formats can still only be converted to open formats on Windows platforms.

#### Identification

As discussed earlier, mass spectral identifications may be based on several factors, but two inputs, the precursor mass (the mass of the molecule) and the fragmentation pattern (through MS/MS) of the precursor mass, are by far the most common identifiers. This spectral information provides a fingerprint unique to most biological molecules; however, low quality spectra cause false positives and false negatives. While improving mass spectrometry will certainly improve spectral quality, improving spectral search algorithms and employing new identification inputs will allow for more confident identifications. This is particularly true for the relatively new fields of metabolomics and lipidomics.

#### Predicting RT

Retention time refers to the amount of time an analyte is delayed by chromatography before exiting and being detected by the mass spectrometer. Retention time is correlated with physical and chemical analyte characteristics; therefore, predicting analyte retention time provides another factor for positive identification. Many peptide retention time prediction strategies exist [38]. However, cross instrument retention times vary greatly due to changes in experimental parameters, creating a real need for retention time normalization as well as retention time prediction.

#### Mass variance correction

Mass variance, the difference between the theoretical and experimental (observed) mass of analytes is an open problem. One way of correcting mass variance is by using the weights of the elements of each analyte to predict m/z locations where a lack of signal is impossible, allowing for the identification of systematic deviation from theoretical masses in a sample [39]. A similar approach is to model such theoretical gaps via a sine curve fitted via a fast Fourier transform [32]. Accurate m/z values are essential to analyte identification.

#### Ontology

According to a recent survey of the field, the biggest problem in lipidomics is the need for a standardization of data acquisition and data processing, due to the huge variability in instruments, protocol and data processing for lipidomics[40]. The many options and permutations in the MS pipeline would make for a very long methods section if explicitly described in a paper--much too long for any journal's page limits. Although several partial ontologies exist (see [41,42]), there is no concise way to uniquely identify an experiment from start to finish, including sample preparation, mass spectrometry protocol, and post-processing. Existing ontologies are particularly lacking in terms of data processing terms. TODO cite clarity in concepts

#### Absolute quantitation

MS signal intensity is related to but not equivalent to analyte quantity [43,44]. Factors that influence this discrepancy include [45]:

• *Ionization efficiency*. Not all analytes in a sample are ionized.

• *Enzyme digestion rate*. When an enzyme--such as trypsin--is used to digest proteins into peptides, not all proteins are completely cleaved. This leads to less-than-expected signal abundance, as the true abundance will be diminished by whole proteins (which are not ionized and therefore not detected), and incompletely digested proteins (which will be detected at different m/z than the expected peptide components).

• *Ion suppression*. When the quantity of analyte entering the ionization mechanism at a given time exceeds the ionization capacity of the ionization mechanism, only a portion of the analyte is charged [46].

Accurate models of these effects would improve estimates of analyte population in samples, as well as further advance *in silico *simulation.

Currently, quantification methods generally fall into one of three approaches: label free spectral counting, quantification via differential stable isotopes, and label free quantification based on the precursor ion signal intensities [47]. Spectral counting is a method in which peptide signals are used to create a protein tally--the count of every protein containing a certain peptide is incremented each time one of its peptides is identified via MS/MS. Despite its prevalence, the accuracy of spectral counting is limited by its dependence on MS/MS acquisition rates, which, as mentioned above, are very low, and its propensity for false positives, since all proteins containing each detected peptide are considered as present when in reality only one need be. Stable isotope labeling methods (SILAC, ICAT, iTRAQ, and TMT) also have significant limitations (see [48]). Besides cost and sample prep complications, nearly all methods increase the number of co-eluting analytes, creating a bottleneck for the complexity of samples handled. What's more, because stable isotope methods target a small specific list of analytes *a priori*, they are not practical in terms of time and money for data-driven discovery, where sample composition is unknown [49].

#### Modeling dynamic range suppression effect

**Figure 8:**
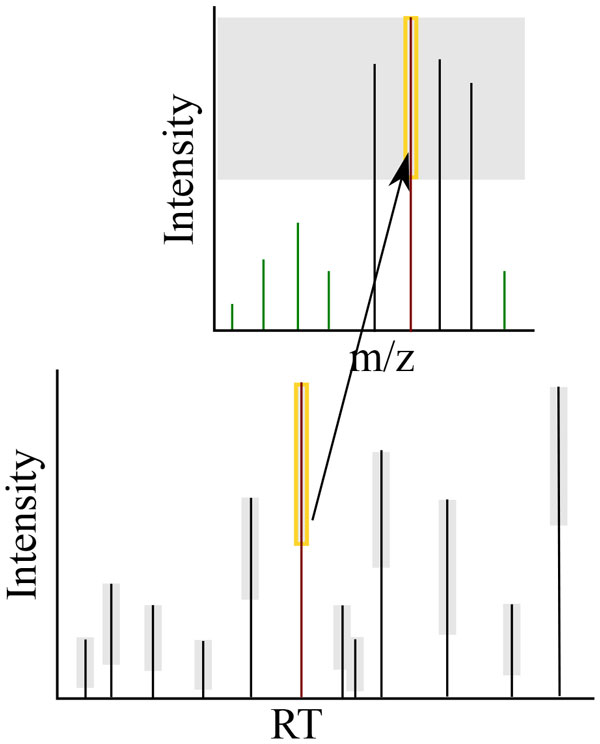
**Dynamic range**. Dynamic range is the window of intensities visible to the sensor at any given RT. The main chromatogram shows the signal of maximum intensity for each RT. The gray box indicates the dynamic range at that RT. The red peak is shown with the other signals at that RT. Note the green peaks will not be detected by the mass spectrometer because they lie outside the dynamic range.

Dynamic range is a term that describes the minimum intensity of a detectable signal given a co-eluting analyte of a higher intensity (see Figure 8). All mass spectrometers have a dynamic range limitation. The current state of the art is 10^3 ^- 10^4^, meaning that at a given RT if one analyte has an intensity of 1.3 × 10^5^, any analyte with an intensity less than 1.3 × 10^2 ^would not be detected.

#### Fragment ion intensities

Because MS/MS acquisition captures not just the analyte of interest but also any surrounding precursor ions, and because fragmentation isn't a perfect process, fragment ion intensities are not as accurate as desired [50,4]. Several machine learning approaches have been proposed for making more accurate fragment identifications [51,52]. However, this is still an open problem.

#### De novo *peptide sequencing*

*De novo *sequencing is an alternative method to database matching that accommodates peptides that don't match up with the database (caused by mutations, polymorphisms, modified amino acids or simply a missing database entry) [53]. Here, the original peptide sequence--defined by a series of letters, each representing an amino acid--is reconstructed based on the MS/MS fingerprint and the chemical properties of the analytes. A recent tutorial provides more detail and resources [54].

#### Fragmentation patterns for lipids

Proteins have a known cleavage pattern, meaning that when peptides are fragmented by MS/MS, association to a peptide is straightforward. Lipids, on the other hand, have a much more complex form due to a wider vocabulary of building blocks and a more complicated fragmentation pattern. To date, no fragmentation rules have been published, making MS/MS much less helpful in lipidomics than proteomics. Because of the complexity of lipids, a machine learning approach could be appropriate in finding a solution to this problem.

#### Biomarker detection

**Figure 9:**
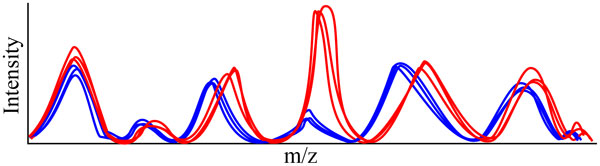
**Difference detection**. Comparative or differential MS-omics aims to identify possible differences between two sets of replicate studies. In this case the three red signals are cases--samples from individuals of interest--and the blue signals are controls--samples from baseline individuals. The center peak clearly indicates a differentially expressed analyte.

Biomarker discovery is the use of comparative analysis (see Figure 9) in order to identify analytes that correlate with certain diseases or other conditions for diagnostics or drug development. It is an active area of research with a lot of published work; however the problem is still wide open due to limitations in mass spectrometry, preprocessing, and identification. Current methods struggle to highlight case/control differences in complex samples, requiring painstaking, time consuming, and error-prone manual detection.

#### Deisotoping

Deisotoping is the process of reducing several instances of the same analyte at different charge states into a single feature--usually a monoisotopic peak (see Figure 3). This is necessary because the query to a data base search consists of only the single-charged feature m/z and (optionally) RT. Adding to the complexity of registering differently charged versions of the same analyte is the fact that, in complex samples, the isotopic envelopes of different analytes can and do overlap, requiring deconvolution (see below).

#### Deconvolution

**Figure 10:**
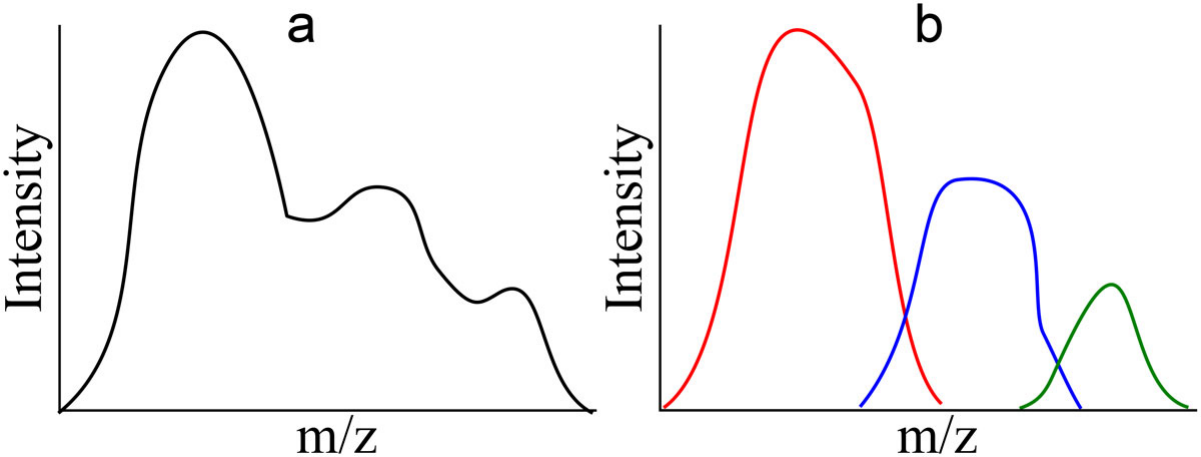
**Deconvolution**. Overlapping analytes create convoluted signals, which must be deconvoluted. This example depicts how three convoluted peaks in profile mode might look in the output of a low resolution mass spectrometer (a). In order to further process the data, they must be deconvoluted into their respective peaks (b).

Overlapping signals must be resolved prior to quantification (see Figure 10). RT overlaps occur when two isobaric analyte elute without a gap between them, and are more common in complex samples. Isotopic envelope overlaps occur in m/z where two analyte are not sufficiently separate in m/z at their current charge state. Ion overlaps occur when particular ions of two given analyte are too similar to be resolved in m/z. All m/z overlaps are less likely in high resolution machines, which by definition are capable of better resolving power evinced by more narrow signals in m/z. RT overlaps can be minimized to some extent by sample preparation and protocol designed to separate similar molecules into different RT areas.

#### Parameter reduction

In general, most algorithms require the user to optimize a host of parameters through manual tuning, which is time intensive. New algorithms should avoid free parameters. If included, they should also provide guidance or an automated method to fix them. Research opportunities include developing methods for automatically optimizing parameters on existing and popular methods.

## Conclusions

MS-omics is an exciting, developing field with many research opportunities for mathematicians, computer scientists, and statitisticians. Although contribution to the field requires a functional understanding of many domain-specific concepts and terms, the open nature of most of the existing problems provides many opportunities for impact.

## Competing interests

The authors declare that they have no competing interests.

## Authors' contributions

RS, ADM, DV, and JTP all contributed in writing this manuscript.

## Declarations

RS is supported by the NSF graduate research fellowship (DGE-0750759).

This article has been published as part of *BMC Bioinformatics *Volume 15 Supplement 7, 2014: Selected articles from the 10th Annual Biotechnology and Bioinformatics Symposium (BIOT 2013). The full contents of the supplement are available online at http://www.biomedcentral.com/bmcbioinformatics/supplements/15/S7
